# The mRNA‐binding proteome of a critical phase transition during Arabidopsis seed germination

**DOI:** 10.1111/nph.17800

**Published:** 2021-11-13

**Authors:** Nikita Sajeev, Anirban Baral, Antoine H. P. America, Leo A. J. Willems, Rémy Merret, Leónie Bentsink

**Affiliations:** ^1^ Laboratory of Physiology Wageningen Seed Science Centre Wageningen University Wageningen 6708PB the Netherlands; ^2^ BU Bioscience Wageningen Plant Research Wageningen 6700 AP the Netherlands; ^3^ Laboratoire Génome et Développement des Plantes CNRS‐LGDP UMR 5096 Perpignan 66860 France

**Keywords:** Arabidopsis, germination, mRNA, RNA binding proteins, seeds, translation

## Abstract

- *Arabidopsis thaliana* seed germination is marked by extensive translational control at two critical phase transitions. The first transition refers to the start of hydration, the hydration translational shift. The second shift, the germination translational shift (GTS) is the phase between testa rupture and radicle protrusion at which the seed makes the all or nothing decision to germinate.
- The mechanism behind the translational regulation at these phase transitions is unknown. RNA binding proteins (RBPs) are versatile players in the post‐transcriptional control of messenger RNAs (mRNAs) and as such candidates for regulating translation during seed germination.
- Here, we report the mRNA binding protein repertoire of seeds during the GTS. Thirty seed specific RBPs and 22 dynamic RBPs were identified during the GTS, like the putative RBP Vacuolar ATPase subunit A and RBP HSP101. Several stress granule markers were identified in this study, which suggests that seeds are prepared to quickly adapt the translation of specific mRNAs in response to changes in environmental conditions during the GTS.
- Taken together this study provides a detailed insight into the world of RBPs during seed germination and their possible regulatory role during this developmentally regulated process.

## Introduction

Seed germination is a complex process in which the seeds need to undergo developmental transitions to successfully establish themselves as a plant. The majority of our understanding on how plant development is regulated has been a product of studying gene expression with the main focus on transcription and DNA binding partners. However, recent studies have highlighted that translational regulation plays an important role in regulating plant development (Sorenson & Bailey‐Serres, 2014; Merchante *et al*., 2017; Sablok *et al*., 2017; Cho *et al*., 2018, 2019; Jang *et al*., 2019). The complete switch‐off state of translation between seed maturation and seed germination makes seeds a unique system to study developmentally regulated translation (Sajeev *et al*., 2019). Previously, it has been shown that there is extensive translational control at two temporal shifts during seed germination. These shifts were defined as the hydration translational shift (HTS) and germination translational shift (GTS) (Bai *et al*., 2017). Interestingly these shifts coincide with important developmental phase transitions during seed germination. The HTS spans the first 6 h after imbibition (HAI); the phase at which seeds take up water. Upon imbibition, the dry seed undergoes a drastic transition from a metabolically inactive to a highly active state. The GTS is the developmental phase between seed testa rupture (TR) and radicle protrusion (RP). These phases mark critical physiological stages of seed germination. Upon TR, seeds can still be dried back without hampering its viability which becomes more difficult as germination progresses. This is because desiccation tolerance can be re‐introduced into seeds only within a limited time frame which is usually lost once upon RP (Maia *et al*., 2011). This developmental transition can be viewed as a point of no return, also known as germination *sensu strictu* (Perino & Côme, 1991). The decision to germinate is based on a complex web of environmental and developmental signals to ensure seedling survival. At the GTS, distinct subsets of messenger RNAs (mRNAs) show differential translation which suggests dynamic regulation of germination (Bai *et al*., 2017). The mechanism behind this selection is yet to be understood. In recent years, several studies have implicated RNA binding proteins (RBPs) can regulate their target mRNAs co‐ and post‐transcriptionally, thereby altering its translation efficiency in plants (Köster *et al*., 2017; Lou *et al*., 2020). Furthermore, a recent study reported that certain stored mRNAs in the dry seed are associated with single ribosomes and RBPs which are later translationally upregulated during germination (Bai *et al*., 2020). This led to the hypothesis that certain RBPs could play a role in determining the fate of the regulated mRNAs during seed germination.

Defining features of RBPs are their putative RNA binding domains (RBDs) like the Pumilio (PUM) domain, Zinc finger domains, K homology (KH) domain or the RNA recognition motif (RRM) (Lorković, 2009). Several studies have demonstrated the role of RBPs in plant development. Some examples include the RBP JULGI that regulates phloem differentiation by translational control of *SUPPRESSOR OF MAX2‐LIKE1‐4/5 (SMXL4/5*) and *ETHYLENE INSENSITIVE2 (EIN2)* a noncanonical RBP that can regulate hypocotyl elongation by repressing the translation of ethylene responsive mRNAs (Merchante *et al*., 2015; Cho *et al*., 2018, 2019). In seeds, through a transcriptomics study, an RBP belonging to the PUM family, *ARABIDOPSIS PUMILIO (APUM) 9* was shown to play a role in delaying seed germination (dormancy) (Xiang *et al*., 2014). Although, recent advancements in RNA‐protein interactome capture techniques have allowed the identification of classical and novel RBPs in different plant tissues, their identity and role in seeds has not yet been explored (Marondedze *et al*., 2016; Reichel *et al*., 2016; Zhang *et al*., 2016; Köster *et al*., 2017; Cho *et al*., 2019; Bach‐Pages *et al*., 2020).

In the present study, mRNA interactome capture was performed in Arabidopsis embryos at TR and RP, the physiological stages that mark the GTS. Hundreds of high confidence RBPs were identified. Additionally, dynamic RBPs were identified in this study like the putative RBP Vacuolar H+‐ATPase subunit A and known RBP HEAT SHOCK PROTEIN 101 (HSP101). These RBPs were also exclusively identified in the seed mRNA interactome capture and not in leaves, protoplasts or etiolated seedlings (Marondedze *et al*., 2016; Reichel *et al*., 2016; Zhang *et al*., 2016; Bach‐Pages *et al*., 2020). Overall, this study provides a valuable resource for future RBP research in seeds and will be the starting point of identifying their possible regulatory role in translation during seed germination.

## Materials and Methods

### Plant materials

Fully after ripened seeds of *Arabidopsis thaliana* accession Columbia‐0 (Col‐0) were used for all assays described in this manuscript (NASC N60000). The mutant line of Hyaluronan/mRNA binding protein (AT5G47210) was obtained from NASC (SALKseq_055953). The complementation lines contain the genomic fragment (forward primer AGGAGGAGGAGGAGAGAA and reverse primer: TCGCAGAAAAGACCTTCA) with its native promoter transferred to the mutant backgrounds using entry vector pDONR207 and the destination vector pKGW‐RedSeed (https://gatewayvectors.vib.be/collection/pkgw‐redseed). The pPABP2‐PABP2‐RFP reporter lines in wild‐type and *hsp101* background were described in Merret *et al*. (2017). The pUBQ‐DCP5‐GFP like was a kind gift from the laboratory of Scheer, Hélène (Scheer *et al*., 2021) while the *hsp101* mutant and complementation line used for the germination phenotypes of Supporting Information Fig. S5 (see later) were a kind gift from Elizabeth Vierling (McLoughlin *et al*., 2019).

### Germination condition and assays

Seeds were sowed on two layers of blue blotter paper (Anchorpaper Co., www.seedpaper.com) were equilibrated with 48 ml of demineralized water in plastic trays (15 cm × 21 cm). Each replicate contained 1.2 g of seeds which were wrapped in a closed transparent plastic bag and placed at 22°C in continuous light (143 µm m^−2^s^−1^) for germination. The time‐points for the GTS were selected based on the physiological stage of the seeds described previously (Bai *et al*., 2017). In this study TR occurred at 26 HAI and RP at 42 HAI.

To determine the DSDS50 values (days of seed dry storage to reach 50% germination), germination assays were carried from 3 d until 5 wk after harvest, when the seeds were fully after‐ripened (100% germination). The germination experiments were performed as described earlier, however at 26°C instead of at 22°C, since these suboptimal germination conditions allowed to also identify smaller differences in dormancy level (Alonso‐Blanco *et al*., 2003). The germination percentages were calculated using the germinator software package (Joosen *et al*., 2010) and the DSDS50 levels were calculated using the statistical program R v.2.14 (R Development Core Team, 2009; www.r‐project.org) (He *et al*., 2014).

### Embryo isolation and ultraviolet crosslinking

For embryo isolation, the imbibed seeds were scraped from the tray and pressed between two microscope slides. Due to the pressure applied, the embryos were expelled out of the seed coat. The embryo‐seed coat mixture was separated in a 40% sucrose solution. Using centrifugation, the mixture was separated and the top layer containing the pure embryos was collected. The embryos were spread evenly over a germination tray containing white Whatmann filter papers to absorb the sucrose solution (L. L.‐M. Lopez‐Molina, pers. comm.).

For *in vivo* crosslinking (CL), the trays were placed on ice and irradiated in a Stratalinker (Stratagene) with 254 nm ultraviolet (UV) light at 1 J cm^−2^. The CL was performed twice with 30 s pause in between treatments. The controls were processed simultaneously. The embryos were harvested immediately after irradiation and frozen in liquid nitrogen (N_2_).

The frozen embryo tissue was ground into fine powder in liquid N_2_ and resuspended in tubes with 24 ml of a modified seed RBP extraction buffer (1.25% sucrose, 400 mM Tris‐HCl pH 8, 0.5% LiDS, 200 mM LiCl, 35 mM MgCl_2_, 1 mM EGTA, 5 mM DTT, 20 U ml^−1^ RNasin, 1× EDTA‐free complete protease cocktail inhibitor tablet). The tubes containing the lysate were placed on ice for 10 min following which they were centrifuged for 20 min at 17 000 ***g*** to precipitate the cell debris. The supernatant (20 ml) from each tube was transferred to fresh RNase free tube. Aliquots from the lysate were taken for quality controls (silver stain, Western blots) and for mRNA enrichment check.

The mRNA–protein complexes were isolated using 1.5 ml of oligo(dT)25 magnetic beads (New England Biolabs, Ipswich, MA, USA) per tube. The beads were equilibrated using 5 ml of wash buffer 1 (20 mM Tris‐HCl pH 7.6, 0.1% LiDS, 500 mM LiCl, 1 mM EDTA, 5 mM DTT) and incubated for 2 min with gentle rotation at 4°C. The tubes were placed on the magnetic rack, which resulted in the magnetic capture of the beads and a clear suspension. Thereafter the supernatant of the magnetic beads was discarded and the cell lysate was immediately added to the tubes and incubated at 4°C for 1 h by applying gentle rotation. Beads were collected on the magnet and washed twice with 15 ml of ice‐cold wash buffer 1, buffer 2 (20 mM Tris‐HCl pH 7.6, 500 mM LiCl, 1 mM EDTA, 5 mM DTT) and wash buffer 3 (20 mM Tris‐HCl pH 7.6, 200 mM LiCl, 1 mM EDTA, 5 mM DTT) for 5 min at room temperature. Finally, the beads were incubated with 500 µl of elution buffer (20 mM Tris‐HCl pH 7.6, 1 mM EDTA) at 50°C for 3 min to release the poly(A)‐tailed RNAs from the beads. Two additional rounds of pulldown were performed for each sample, and the three eluates were combined in a new RNase free tube (total volume 1.5 ml).

### Messenger RNA enrichment check using qRT‐PCR

Aliquots taken of the total input and after poly‐A pulldown samples were spiked with a mix of the four eukaryotic poly(A) RNAs (Affymetrix, Santa Clara, CA, USA; P/N900433; Ambion), and purified with TriPure Isolation Reagent (Roche, Basel, Switzerland). Complementary DNA (cDNA) was synthesized using the iScript™ cDNA synthesis kit (Bio‐Rad, Hercules, CA, USA) according to the manufacturer's protocol. Quantitative reverse transcription polymerase chain rection (qRT‐PCR) was performed using Power SYBR Green (Applied Biosystems, Waltham, MA, USA) in a 10 μl reaction using the standard program of the ViiA™ 7 instrument (Applied Biosystems). To quantify RNA levels, the comparative *C*_t_ method, namely the $2^{‐\Delta \Delta C_{t}}$ method was used and normalized to the geometric mean of the spike‐in standards (Livak & Schmittgen, 2001).

### RNA quantification and normalization for SDS‐PAGE loading

The pooled eluates were quantified using the NanoDrop spectrophotometer (260 : 280 ratios between 1.7–2.0). All samples were normalized for mRNA quantity in both time‐points and for each replicate using the elution buffer.

### RNase treatment and protein concentration

The mRNA was digested by adding 100 units of the commercially available RNase cocktail containing RNase A and T1 to the eluates. The samples were mixed and incubated at 37°C for 1 h along with a negative control sample. After the RNase digestion the samples were concentrated using Amicon® centrifugal filter units (0.5 ml, 3 kDa). Each sample was concentrated to approximately 40 µl in low‐binding Eppendorf tubes.

### SDS‐PAGE, silver staining and immunoblot

Briefly, 20 µl of the concentrated protein samples mixed with 5× sodium dodecyl sulphate (SDS) loading dye were loaded on a 12% Bis‐Tris protein gel (Thermo Fisher Scientific, Waltham, MA, USA). The gel was run at 100 V until the loading dye reached the end of the resolving gel. The sodium dodecyl sulphate–polyacrylamide gel electrophoresis (SDS‐PAGE) gel was washed twice with ultra‐pure water for 5 min each time. The silver staining was performed using liquid chromatography–tandem mass spectrometry (LC–MS/MS) compatible silver staining protocol (Mortz *et al*., 2001).

For Western blotting, following the SDS‐PAGE, the gels were electroblotted on to polyvinylidene fluoride (PVDF) membranes (Trans‐Blot Turbo Mini 0.2 μm PVDF transfer packs; Bio‐Rad). The membranes were blocked with 5% nonfat milk in 1× TBST (1× TBS with 0.1% Tween 20) for 1 h at room temperature, followed by an overnight incubation at 4°C with primary antibodies in 3% nonfat milk with rotation. The membrane was incubated with secondary in 3% nonfat milk in 1× TBST for 1 h at room temperature. Protein signals were detected using a high sensitivity enhanced chemiluminescence (ECL) substrate and visualized using the Chemidoc (Bio‐Rad). The primary antibodies used were Anti‐AGO1 (AS09‐527; Agrisera, Vannas, Sweden), Plant Anti‐Actin (AS13 2640; Agrisera), Anti‐HSP101 (a kind gift from Elizabeth Vierling, Amherst, MA, USA) and Anti‐V‐ATPase subunit A (AS09467; Agrisera). The secondary antibody used was horseradish peroxidase (HRP)‐conjugated Anti‐Rabbit immunoglobulin G (IgG) concentrate (Item I1) (RABHRP1; Sigma Aldrich).

### Sample preparation for proteomics

The gel lanes were cut out per sample. The lanes were cut such that it did not include the RNAse enzyme bands present in the lane. Each lane was cut into tiny pieces and divided equally over three Eppendorf tubes. The gel pieces were washed with milliQ water and 100% acetonitrile (ACN). For reduction and alkylation, the gel pieces were incubated with 100 µl of 10 mM DTT in 50 mM ammonium bicarbonate (pH 7.6) at 56°C for 45 min. The samples were brought back to room temperature. Supernatant was removed and gel pieces were washed with 50% ACN. Following this, 100 µl of 54 mM iodoacetamide was added to the gel pieces. The tubes were incubated at room temperature for 20 min in the dark. The gel pieces were washed three times using 100% ACN and ammonium bicarbonate alternatively. After the last wash with ACN, the gel pieces were shortly dried on air and then incubated overnight with 10 ng of trypsin in 50 mM ammonium bicarbonate (pH 7.6) at 37°C for protein digestion. The next day the peptides were extracted from the gel twice with 50 µl 50% ACN and 100% ACN. Next, the pooled extracts were vacuum dried for 2 h and the dried pellets were dissolved in 0.1% formic acid and used for MS.

For the input total protein samples, 50 µg of the lysate was used in a total volume of 25 µl. Following this step 3 µl of iodoacetamide was added to lysate and incubated at room temperature for 20 min in the dark. Next, 3 µl of 12% phosphoric acid was added to the sample. To prepare lysates that contain detergents like lithium dodecyl sulphate for nano‐LC–MS/MS, the S‐Trap™ Micro spin columns = digestion protocol was used (https://protifi.com/pages/s‐trap) according to the manufacturer’s protocol. An overnight column protein digestion was performed on the samples, using 1.5 µg of trypsin in 50 mM ammonium bicarbonate (pH 7.6) at 37°C. The next day the peptides were eluted from the columns with 35 µl of 50% ACN and 0.2% formic acid. Next, the pooled extracts were vacuum dried for 2 h and the dried pellets were dissolved in 0.1% formic acid and used for MS.

### Liquid chromatography–tandem mass spectrometry analysis

Samples were analysed on an LTQ‐Orbitrap Velos Pro mass spectrometer (Thermo Fisher Scientific) coupled to a nanoAcquity UPLC system (Waters, Milford, MA, USA). Peptides were loaded onto a trapping column (nanoAcquity Symmetry C18, 5 μm, 180 μm × 20 mm) at a flow rate of 15 μl min^−1^ with solvent A (0.1% formic acid). Peptides were separated over an analytical column (nanoAcquity BEH C18, 1.7 μm, 75 μm × 200 mm) at a constant flow of 0.3 μl min^−1^ using the following gradient: 3% solvent B (ACN and 0.1% formic acid) for 10 min, 7 to 25% solvent B within 210 min, 25 to 40% solvent B within 10 min, and 85% solvent B for 10 min. Peptides were introduced into the mass spectrometer using a Pico‐Tip Emitter (360 μm outer diameter × 20 μm inner diameter, 10 μm tip; New Objective Inc., Littleton, MA, USA). MS survey scans were acquired from 300 to 1700 *m*/*z* at a nominal resolution of 30 000. The 15 most abundant peptides were isolated within a two‐dimensional (2D) window and subjected to MS/MS sequencing using collision‐induced dissociation in the ion trap (activation time, 10 ms; normalized collision energy, 40%). Only 2+/3+ charged ions were included for analysis. Precursors were dynamically excluded for 30 s (exclusion list size was set to 500).

### Peptide and protein Identification

Raw data were processed using MaxQuant (v.1.6.1) (Cox & Mann, 2008). MS/MS spectra were searched against the Araport11 Arabidopsis database (input proteome v. 11/07/2015 including 50.164 entries) concatenated to a database containing protein sequences of common contaminants. Enzyme specificity was set to trypsin/P, allowing a maximum of two missed cleavages. Cysteine carbamidomethylation was set as fixed modification, and methionine oxidation and protein N‐terminal acetylation were used as variable modifications. The minimal peptide length was set to six amino acids and a minimum of one unique peptide was required for the identification. The mass tolerances were set to 20 ppm for the first search, 6 ppm for the main search, and 0.05 Da for product ion masses. False discovery rates (FDRs) for peptide and protein identification were set to 1%. Match between runs (time window 2 min) and requantify options were enabled, as well as the IBAQ function.

### Definition of GTS‐RBPs and candidate RBPs

The proteinGroups.txt output from MaxQuant was further processed in Perseus v.1.6.12 from MaxQuant (Tyanova *et al*., 2016). Proteins that were identified in at least two or more biological replicates of the CL treatment and with a minimum of two unique peptides identified the proteins were selected for further analysis. To be able to perform statistics between the noncrosslinking (NCL) and CL samples, all normalized label‐free quantitation (LFQ) intensities were log_2_ transformed and the missing values were replaced by a constant minimum value of 10. Next, *t*‐tests were performed with a Benjamin–Hochberg correction for multiple *t*‐testing and a FDR of 5% between the NCL and CL for each stage. Proteins that were statistically enriched in the CL samples were defined as the GTS‐RBPs per time‐point and the ones that were not statistically enriched but had a log_2_ fold (CL/NCL) ≥ 1, were defined as the candidate RBP set per stage. Similar analysis was performed for the input total protein. Here only the LFQ intensities of all proteins identified in the CL input total protein samples were compared between the TR and RP stages.

### Gene ontology (GO) and Pfam annotation and analysis

Gene ontology and Pfam annotation for the proteins was performed using the Perseus tool (v.1.6.12) (Tyanova *et al*., 2016) using the GO database and Pfam database plugins. Proteins that contained the term RNA binding in their GO annotation were categorized as the ‘RNA binding’ set. Pfam classification was done on the RNA binding and Not binding set by counting the number of proteins per protein family in each stage. The proteins were classified as classical or nonclassical RBPs based on previous reports. GO enrichment analysis was performed using the g:Profiler tool (http://biit.cs.ut.ee/gprofiler/) (Raudvere *et al*., 2019) using the Arabidopsis genome as a reference dataset. For statistical *t*‐tests, Benjamin–Hochberg correction for multiple testing was chosen with 0.05 as the significance level.

### Confocal image analysis

For visualization of all reporter lines used in this study, epidermal cells from embryonic root tips (at TR and RP stages imbibed in water) were imaged with a Leica SP8 laser scanning confocal microscope equipped with ×63 oil immersion objective (NA 1.4). For the heat stress treatment, embryos were excised and exposed to a short heat stress of 42°C in water for 30 min before loading onto a slide for visualization under the Leica SP8 confocal microscope. Yellow fluorescent protein (YFP) and red fluorescent protein (RFP) fluorophores were excited with 488 nm and 552 nm laser lines, respectively, and their fluorescence emissions were collected in 515–550 nm and 580–650 nm windows, respectively. For each category, 30 epidermal cells from five seedlings were measured (*n* = 30). The number of granules were quantified using ImageJ plugin three‐dimensional (3D) object counter (Du *et al*., 2011). Maximum intensity of a Z projection covering a depth of 5 µm deep from the cell surface was quantified. Particles within a diameter range of 20 to 100 pixels were measured. The data was plotted as number of granules per 1000 µm^3^ volume, the data normality was checked by Kolmogorov–Smirnov test and variance equality was checked by Levene’s test.

## Results and Discussion

### Identification of the mRNA binding proteome at the germination translational shift

**Fig. 1:**
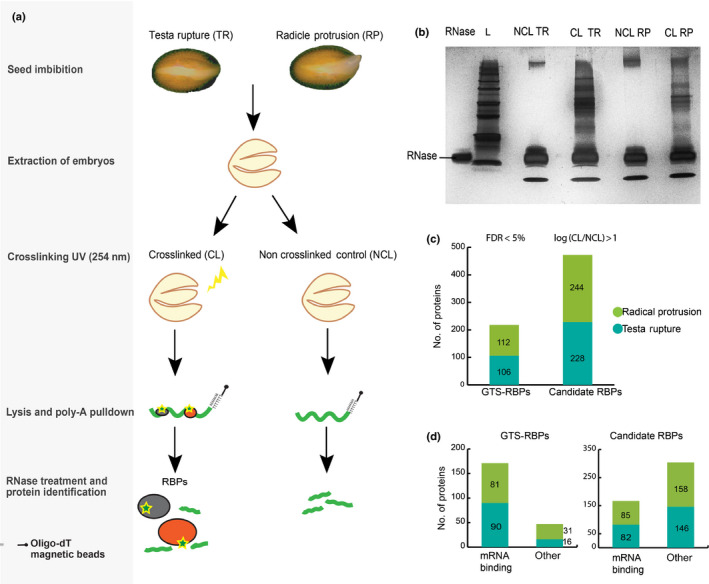
Messenger RNA (mRNA) interactome capture of the Arabidopsis germination translational shift (GTS). (a) Schematic representation of mRNA interactome capture at testa rupture (TR) and radicle protrusion (RP), the two stages that define the GTS. (b) A silver stained SDS‐PAGE gel showing the RNAse enzyme control at the left side of the protein ladder (L) and to the right side are the mRNA–protein complexes that were isolated from the noncrosslinking (NCL) and crosslinking (CL) samples of the TR and RP stages. Results are representative of three independent interactome capture experiments with three biological replicates. (c) Bar graphs representing GTS‐RBPs (RNA binding proteins) which were proteins identified with high confidence false discovery rate (FDR) < 0.5 and candidate RBPs that show log_2_ (CL/NCL) > 1 enrichment at TR and RP. (d) Categorization of the GTS‐RBPs and candidate RBPs based on the gene ontology term ‘RNA binding’.

The GTS defines the period of translational regulation between TR and RP. The exact moment of RP is genotype and environment dependent, which implies that this has to be determined for every new experiment. In this experiment, TR and RP occurred at 26 and 42 HAI for Arabidopsis ecotype Col‐0 seeds (Fig. S1). To unravel the mRNA binding proteome during the GTS, the existing mRNA binding interactome protocol had to be extensively adapted for Arabidopsis embryos (Castello *et al*., 2013) (Fig. 1a). The mRNA interactome capture was performed on the embryos of three independent biological replicates at TR and RP. To summarize, UV radiation was used to crosslink the mRNA–RBP complexes while processing the noncrosslinked controls in parallel. The embryos were lysed in a denaturing buffer and poly‐A mRNA was pulled down using oligo‐dT magnetic beads (Fig. 1a). Poly‐A mRNA enrichment was seen in the eluates after poly‐A pulldown compared to the total input RNA before pulldown using qPCR (Fig. S2). Next, the enrichment of proteins in the CL samples over the NCL was confirmed using silver stained SDS‐PAGE gels (Fig. 1b). The samples were then analysed using label free nano‐LC–MS/MS analysis. Scatter plots of the LFQ intensities between the replicates showed good reproducibility at both time‐points (Fig. S3).

Over 1300 proteins were identified across all samples. However, only proteins for which two or more unique peptides were detected in at least two biological replicates of the CL samples were taken for further analysis. This resulted in more than 600 proteins that were enriched in the CL samples in both stages. Thus, 106 and 112 proteins were identified as high confidence RBPs (FDR < 5%) at TR and RP, respectively (GTS‐RBPs), with an overlap of 54 proteins that were present at both time‐points (Table S1a,b). Although, several proteins did not pass these stringent parameters of selection, many proteins were highly enriched in the crosslinked samples over the controls and therefore could be important RBPs that play a role in the GTS. Hence, 228 proteins at TR and 244 proteins at RP with a log_2_ fold (CL/NCL) enrichment > 1 were classified into a second set called the candidate RBPs for each time‐point in our dataset (Fig. 1c; Table S1a,b).

Next, the GTS‐RBPs and candidate RBPs were annotated based on their molecular function. This revealed that approximately 80% of the GTS‐RBPs had been previously annotated with known or predicted RNA binding activity, while 47 GTS‐RBPs were not and could be putative RBPs (Fig. 1d). The candidate RBP set showed a large proportion of RBPs not annotated as mRNA binding and therefore provide a repertoire of putative RBPs in seeds (Figs 1d, S1a,b). A GO enrichment analysis for all GTS and candidate RBPs over the two time‐points showed common enrichment for GO terms like binding, mRNA binding, heterocyclic compound binding and organic cyclic compound binding (Table S1c). Overall, the GO analysis, revealed that the interactome capture strongly enriched for proteins related to RNA biology.

### Protein domain analysis reveals stage specific protein families during the GTS

**Fig. 2:**
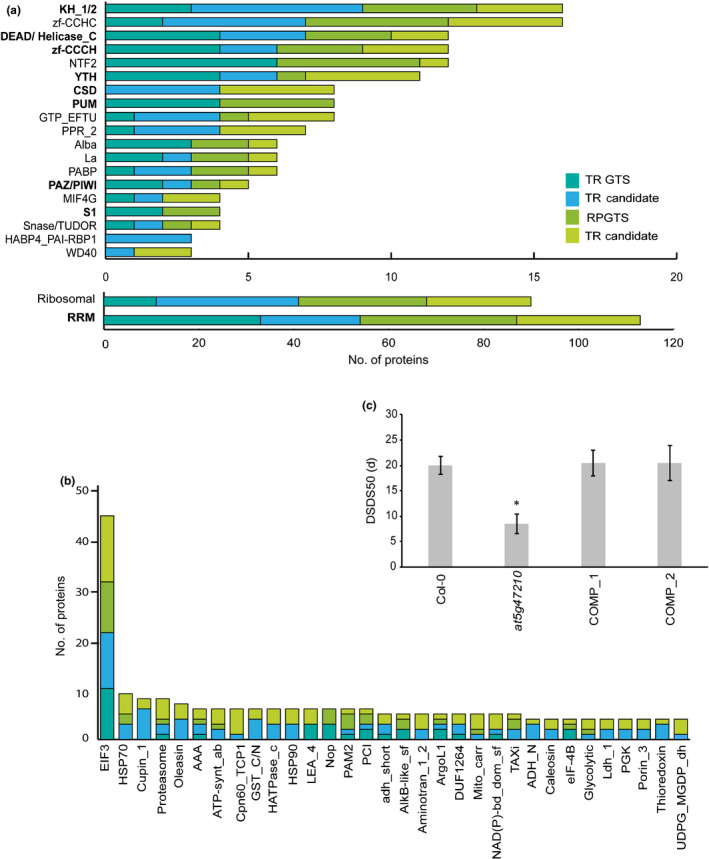
Proteins domain classification of the identified germination translational shift (GTS) and candidate RNA binding proteins (RBPs) in the GTS of *Arabidopsis thaliana*. (a) Classical and nonclassical RNA binding domains (RBDs) at testa rupture (TR) and radicle protrusion (RP). The classical RBDs are indicated in bold (families with ≥ 3 proteins depicted in figure). (b) Putative RBDs at TR and RP (families with > 4 proteins depicted in figure). (c) Graph representing the days of seed dry storage to reach 50% germination (DSDS50). A mutant of the hyaluronan/mRNA binding protein AT5G47210 (*at5g47210*) and two complementation lines (COMP_1 and COMP_2) were analysed for their DSDS50 compared to the wild‐type Col‐0 at 26°C. The results are representative averages of four biological replicates (*t*‐test, asterisk (*) indicates statistical significance *P* < 0.05; error bars represent the SE).

Both the GTS‐RBPs and candidate RBPs at TR and RP were grouped by their protein domain annotations (Pfam or Interpro annotations) (Fig. 2a; Table S1d). At both stages, diverse classical and nonclassical RBDs were captured (Fig. 2a). Examples of classical domains include RRM, KH domain, Zinc finger (zf)‐CCCH, DEAD box Helicases and PUM. The vast majority of the RBPs identified contained the RRM domain (Fig. 2a). The Arabidopsis proteome consists of 253 proteins containing an RRM domain (Lorković & Barta, 2002). The RRM family is highly diverse in plants and in this study 66 GTS‐RBPs and 47 candidate RBPs containing an RRM domain were identified in seeds. Majority of the RRMs have not been investigated for their roles in germination and could be important regulators of germination. An example of such a regulator is an RRM containing glycine rich protein, atRZ‐1a, which was identified as a candidate RBP at RP. This RBP has been reported to negatively impact germination under salt and osmotic stress (Kim *et al*., 2007). The Arabidopsis PUM family contains 25 proteins that are phylogenetically classified into four groups. Interestingly only group 1 APUM RBPs (APUM1,3,5 and 6) were identified as GTS‐RBPs at both stages indicating that group 1 APUMs are especially abundant during seed germination.

Nonclassical RBDs like Ribosomal, La and GTP‐EFTU were also well represented at both TR and RP (Fig. 1a). The nonclassical RBD, HABP4_PAI‐RBP1 family was only identified in the candidate RBP set at TR (Fig. 2a). Three Hyaluronan/mRNA binding proteins contained this RBD namely, AtRGGA, AT5G47210 and AT4G17520. AtRGGA has been reported to play a role in abscisic acid (ABA) signalling during stress response in seedlings. Mutants of this RBP are highly susceptible to salt and osmotic stress (Ambrosone *et al*., 2015). *AT5G47210* was revealed to be highly expressed 1 d after seed imbibition followed by a reduction at later time‐points (Narsai *et al*., 2011). These time‐points closely coincide with the stages of TR and RP and could explain why this protein is no longer identified at RP point. In the present study, one knockout mutant (Fig. S4) and two complementation lines of *AT5G47210* have been investigated for seed germination phenotypes. This revealed a dormancy phenotype, measured as DSDS50 value (Alonso‐Blanco *et al*., 2003; Soppe & Bentsink, 2020). The knockout mutant *at5g47210* had a DSDS50 of only 8.5 d in comparison to its wild‐type Col‐0 which required 20 d (Fig. 2c). The complementation lines COMP1 and COMP2, complemented this mutant phenotype (Fig. 2c). Therefore, AT5G47210 could play a role in inhibiting germination. The mechanism by which this RBP regulates germination needs to be further explored.

The domain analysis also revealed many putative RBDs many of which belonged to the elongation Initiation factor 3 (EIF3) family (Fig. 2b). Other protein families such as HSP70, AAA and DUF1264 have also been identified as putative RBDs in previous studies (Reichel *et al*., 2016; Bach‐Pages *et al*., 2020). Interestingly, many enzyme families like Phosphoglycerate kinase (PGK), thioredoxins, glutathione‐S‐transferase (GST), and NAD(P) binding domain (NAD(P)‐bd_dom_sf) proteins were pulled down in this study (Fig. 2b; Table S1d). There have been more reports on metabolic enzymes with RNA binding functions in eukaryotes (Castello *et al*., 2015; Marondedze *et al*., 2016; Reichel *et al*., 2016; Bach‐Pages *et al*., 2020). PGKs and thioredoxins have been validated as RBPs in humans and yeast cells (Beckmann *et al*., 2015). In plants, it has been shown that GSTs are modulated by atRZ‐1a, an RRM and Zinc finger domain containing protein also identified as a GTS‐RBP in this study. This report concluded that this enzyme among others play a role in ROS homeostasis during germination (Kim *et al*., 2007). In another study, some NAD(P) binding domain proteins were identified as RBPs that respond to osmotic stress (Marondedze *et al*., 2019). Most enzyme families identified in this interactome capture have been known to play a role in reactive oxygen species (ROS) homeostasis. However, their discovery as a putative RBDs in this study, suggests novel roles for these metabolic enzymes as RBPs in the translational regulation of seed germination.

### Dynamic RBPs identified during the GTS

An in‐depth analysis into the nonoverlapping GTS‐RBPs (106 at TR and 112 at RP) showed that many RBPs were identified as a GTS‐RBP at one time‐point and as a candidate RBP in the other. However, only 22 RBPs of these GTS‐RBPs were exclusively identified in one time‐point alone and therefore classified as dynamic GTS‐RBPs (Table S2). The dynamic GTS‐RBPs included known RBPs like EIN2 and HSP101 (Merchante *et al*., 2015; Merret *et al*., 2017). EIN2 mutants have been shown to have a very strong dormancy phenotype due to high ABA levels in the dry seed (Koornneef *et al*., 2002). This study demonstrates that EIN2 can also function as an RBP during seed germination. HSP101 was reported to bind and regulate the translation of the internal light‐regulatory element (iLRE) of ferredoxin (Fed‐1) mRNA in carrot protoplasts (Ling *et al*., 2000). A recent study further showed that HSP101 is required for the efficient release of ribosomal protein mRNAs from stress granules for the rapid recovery of the translational machinery from heat stress (Merret *et al*., 2017). Traditionally, heat shock proteins (HSPs) are regarded as conserved molecular chaperones involved in protein folding stability and activation. However, several other HSPs such as HSP81.2, HSP70 and HSP70b were identified as part of the candidate RBP set at TR while HSP60, HSP91 chloroplast and mitochondria HSP70.1 were identified in the candidate RBP dataset at the RP stage. HSP101 was the only GTS‐RBP identified exclusively at the TR point and could function as an RBP involved in the phase transition from TR to RP, however the *hsp101* mutant did not show a germination or dormancy phenotype compared to wild‐type (Fig. S5).

We also identified many dynamic putative GTS‐RBPs. An example of a dynamic GTS‐RBP with no links to RNA biology is the VACUOLAR H+‐ATPase SUBUNIT A (V‐ATPase SUBUNIT A) identified at the RP stage. V‐ATPases are versatile multi‐subunit proton pumps that control the pH of many intracellular compartments in all eukaryotic cells. In Arabidopsis, V‐ATPases play a role in plant defences against environmental stresses like salt stress. The subunit A gene detected in Arabidopsis can produce at least four different transcripts by using different polyadenylation sites. These transcripts differ only in their 3' untranslated region and produce identical proteins (Magnotta & Gogarten, 2002).

**Fig. 3:**
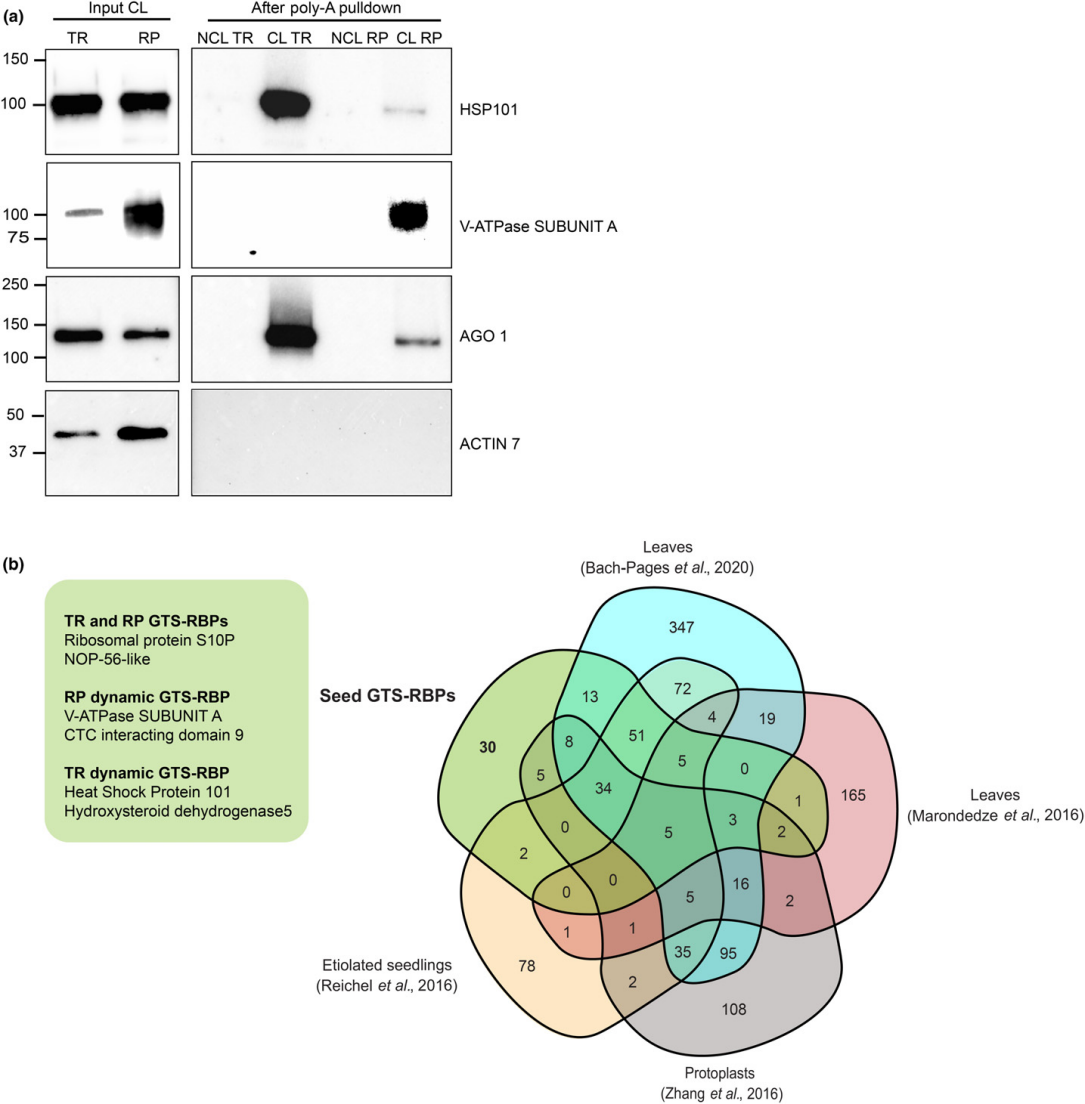
Dynamic germination translational shift‐RNA binding proteins (GTS‐RBPs) and seed specific RBPs identified during the GTS of *Arabidopsis thaliana*. (a) Western blot image confirming the dynamic nature of GTS‐RBPs HSP101 at the testa rupture (TR) stage and V‐ATPase subunit A at the radicle protrusion (RP) stage after the poly‐A pulldown. AGO1 was used as a known RBP control, while ACTIN 7 as a non‐RBP negative control. The noncrosslinking (NCL) and crosslinking (CL) samples were normalized based on the mRNA quantity after the poly‐A pulldown, while the total protein input for the CL samples were loaded with a fixed volume of the total protein from the CL lysates. (b) Venn diagram comparing the GTS‐RBPs identified in this study and previously performed mRNA interactome captures in different plant tissues. The green box shows representative seed specific GTS‐RBPs that were either identified at both stages or dynamic for the TR or RP stages.

The dynamic nature and the RBP identity for HSP101 and V‐ATPase SUBUNIT A was validated using Western blotting (Fig. 3a). ARGONAUTE 1 (AGO1) being a well‐established RBP also identified in this study was used as a positive control and ACTIN 7 was used as a negative control (Fig. 3a). The results confirmed the dynamic nature of HSP101 and V‐ATPase SUBUNIT A which were highly abundant in the CL samples at TR and RP, respectively (Figs 3a, S6). Although AGO1 showed similar LFQ intensities at TR and RP in this study, the Western blot showed some dynamics for this protein indicating the qualitative rather than quantitative nature of label free proteomics. The negative control ACTIN 7 was only present in the total protein of TR and RP and not after the poly‐A pulldown, demonstrating the stringency of the mRNA interactome procedure. To confirm that the changes observed after the interactome capture were not due to differences in total protein abundance, an additional proteomics analysis on the total input protein fractions was performed at both stages. As highly abundant proteins can limit the identification of less abundant proteins, we were able to identify only 11 out of the 22 dynamic GTS‐RBPs in the total input protein samples (Tables S1f, S2). The data confirmed that there were no significant differences in protein abundance for HSP101 and V‐ATPase SUBUNIT A at TR and RP before the interactome capture. This further supports our hypothesis that HSP101 and V‐ATPase SUBUNIT A are dynamic RBPs at TR and RP, respectively.

HSP101 plays a role in releasing ribosomal RNAs from stress granules for heat stress recovery (Maia *et al*., 2011; Merret *et al*., 2017). In the case of V‐ATPase SUBUNIT A, its function as an RBP is unclear. It has been previously reported that vacuoles from tomato protoplasts can contain RNA oligonucleotides (Abel *et al*., 1990) and a recent study demonstrated that, RNAse T2 ribonucleases are targeted to vacuoles for ribosomal RNA degradation and maintenance of cellular homeostasis in Arabidopsis (Floyd *et al*., 2017). Both these studies show that RNAs can be targeted to vacuoles. A plausible hypothesis could be that putative GTS‐RBP V‐ATPase SUBUNIT A is involved in the sequestration of RNA to the expanding vacuoles at RP to maintain cellular RNA homeostasis. However, further research is required to establish the RBP identity and roles of both these RBPs during the GTS.

### Comparison with other plant interactome captures reveal seed specific RBPs

Due to technical advancements in the recent years, mRNA interactome capture has gained a momentum in plant research. In the last five years, four different studies have published the mRNA interactome of Arabidopsis seedlings (300 RBPs), leaves (717 and 230 RBPs) and protoplasts (325 RBPs) (Marondedze *et al*., 2016; Reichel *et al*., 2016; Zhang *et al*., 2016; Bach‐Pages *et al*., 2020). Although the previous studies identified much larger sets of statistically enriched RBPs, a comparative analysis of all GTS‐RBPs identified in this study with previously performed interactome captures revealed 30 GTS‐RBPs that were only identified in germinating seeds and five RBPs that were common to all datasets (Fig. 3b; Tables S1e, S3). This shows that Arabidopsis RBPs are highly versatile, tissue and developmental stage specific. Eleven out of these 30 seed specific GTS‐RBPs had been previously annotated with an mRNA binding function and contained classical RBDs. Many previously unknown RBPs in this set were enzymes like H(+)‐ATPase 1, pyruvate orthophosphate di‐kinase and hydroxysteroid dehydrogenase 5 (Fig. 3b; Table S3). Interestingly, nine out of the 30 seed specific RBPs are also part of the dynamic GTS‐RBPs set identified in this study.

### Stress granule markers enable quick responses to the environment

**Fig. 4:**
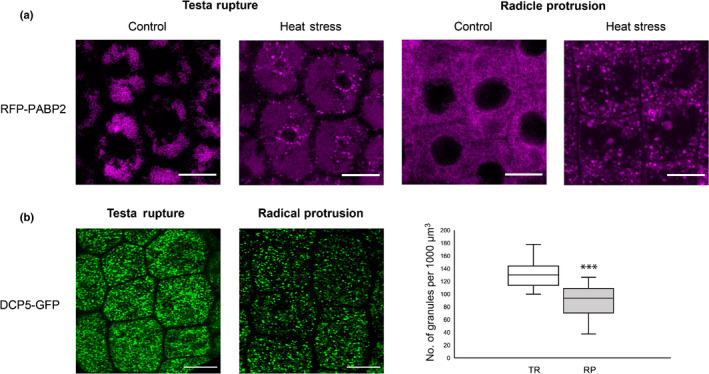
Visualization of P‐bodies and stress granules at the testa rupture (TR) and radicle protrusion (RP) stages of *Arabidopsis thaliana* seed germination. (a) Visualization of stress granules using reporter line pPABP2‐tRFP‐PABP2 at TR (background bodies are large vacuoles commonly present at this stage) and RP at optimal germination conditions (control) or under short heat stress of 30 min at 42°C (b) Visualization of P‐bodies using reporter line pUBQ‐DCP5‐GFP at RP and TR. Box‐plot showing the number of granules within a diameter range of 20 to 100 pixels per 1000 µm^3^ volume (*n* = 30 root epidermal cells and five embryos per stage, Bars, 10 μm; *t*‐test, asterisks (∗∗∗) indicates statistical significance *P* < 0.001). Indicated are the median intensity (middle line), the upper and lower 10^th^ percentile and the whiskers show the range of the data.

During the GTS the seed makes an all or nothing decision to germinate or not. In a biological context, germination must only proceed when the environmental conditions allow the successful establishment of the seedling. Several proteins that have been previously described to be part of cytoplasmic stress granules were identified at both stages with similar LFQ intensities like, RNA BINDING PROTEIN 47 A (RBP47), RBP47B, OLIGOURIDYLATE‐BINDING PROTEIN 1C (AtUBP1c) and POLY‐A BINDING PROTEIN 2 (PABP2). Stress granules are cytoplasmic foci which are formed in response to various environmental stresses like salt stress, hypoxia and heat stress (Chantarachot & Bailey‐Serres, 2018). Stress granules can transiently store mRNAs until the stress resolves, allowing cells to quickly repress the translation of specific mRNAs in a stressful situation. To show that stress granule markers quickly respond to stressful conditions, a reporter line of stress granule marker RFP‐PABP2 was imaged at TR and RP in response to heat stress (Fig. 4b). At control conditions PABP2 was expressed throughout the cytoplasm in the radicle tip of embryos and did not show any clear foci formation. Interestingly, after a short heat stress, PABP2 is clearly localized into cytoplasmic stress granules at both TR and RP (Fig. 4a). Further, to explore whether dynamic GTS‐RBP HSP101 could regulate translation at TR, PABP2 was imaged in *hsp101* background after a short heat stress. (Maia *et al*., 2011; Merret *et al*., 2017). As expected, the number of stress granules in the *hsp101* seeds were significantly higher than wild‐type at the TR stage (Fig. S7) suggesting that HSP101 is a GTS‐RBP that could play a role in the translational control of germination via stress granules. This suggests that seeds during GTS possibly express certain stress granule markers in preparation for a quick adaptation of translation in response to changed environmental conditions.

P‐bodies are also cytoplasmic granules in which translationally repressed mRNAs can be decayed or stored for development or stress responses (Narsai *et al*., 2011; Hubstenberger *et al*., 2017). P‐bodies can contain several RBPs, 5′ to 3′ exoribonucleases, de‐adenylation factors and factors involved in nonsense‐mediated mRNA decay (Maldonado Bonilla, 2014). Some examples of P‐body components are DECAPPING PROTEIN 1 (DCP1), DCP2, DCP5 and EXORIBONUCLEASE4 (XRN4) (Xu & Chua, 2009). Although, many of these P‐body makers are known to be expressed in seeds, we only identified DCP5 as a dynamic GTS‐RBP at the TR point. Previously, DCP5, has been shown to play a role in the translational repression of mRNAs via P‐bodies in seedlings and in dark/light phase translation (Xu & Chua, 2009). To explore the localization of DCP5 during the GTS, a DCP5‐GFP reporter line was imaged at TR and RP (Fig. 4a). At TR, DCP5 forms more cytoplasmic granules than at the RP stage in the epidermal cells of the radicle tip (Fig. 4b). This differential localization could explain why DCP5 was identified as a dynamic GTS‐RBP in the present study. Interestingly, DCP5 was the only well‐established P‐body marker identified in the interactome capture of leaves and in seedlings (Reichel *et al*., 2016; Bach‐Pages *et al*., 2020) while for example, DCP1 was not. This indicates that the mRNA interactome capture method may not be an ideal to pull down all types of cytoplasmic granules. This could be explained by the fact that P‐bodies contain deadenylation factors that degrade the poly‐A tails of the mRNAs and in the present interactome capture, only poly‐A mRNAs were pulled down (Maldonado Bonilla, 2014) or it could be that the mRNAs present in these bodies are not easily accessible to the oligo‐dT beads used in this study.

**Fig. 5:**
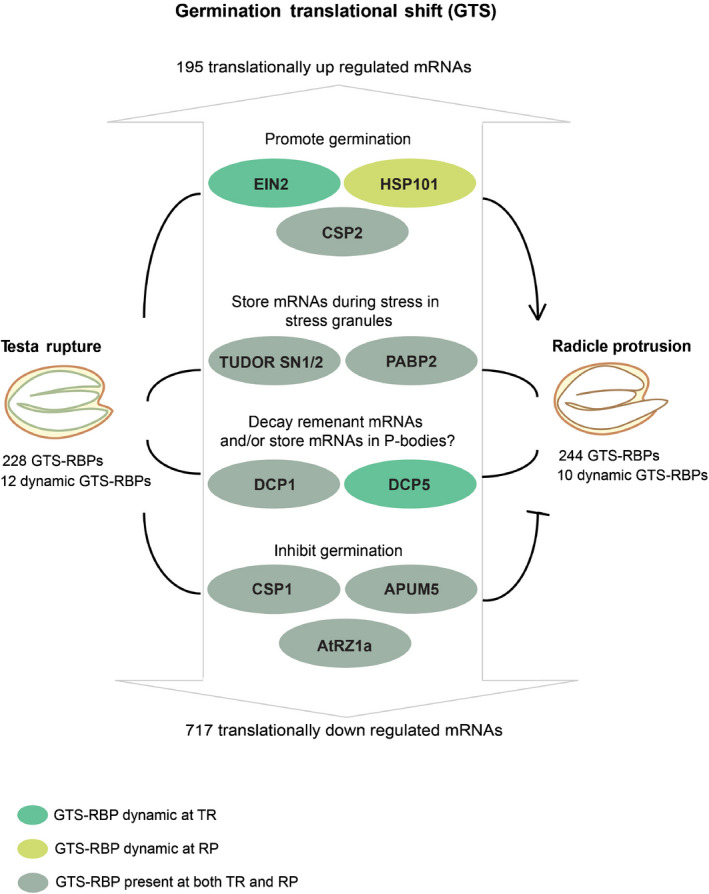
Summary of the features at the germination translational shift (GTS). The number of RNA binding proteins that are identified at testa rupture (TR) and radicle protrusion (RP) are indicated at the left and right side of the figure, respectively. The box in the middle of the figure presents the GTS‐RBPs (RNA binding proteins) (ovals) that play a role in germination and may regulate the translational of messenger RNAs (mRNAs) during the GTS. The arrows indicate proteins that promote germination and flat headed arrows indicate proteins that inhibit germination. Proteins in the middle (TUDORSN1/2, PABP2, DCP1 and DCP5) could either promote or inhibit germination depending on the environmental conditions. The numbers indicated in the top and bottom of the figure represent the mRNAs that are under translational control identified by Bai *et al*. (2017).

In summary, the GTS spans a critical phase during germination at which extensive translational regulation takes place in which 195 and 717 mRNAs are translationally upregulated and downregulated, respectively (Bai *et al*., 2017) (Fig. 5). The mechanism behind this selection is yet to be elucidated. The fate of the regulated mRNAs could be controlled by RBPs present during this shift. Over 600 GTS and candidate RBPs were identified. Among these, 228 and 244 GTS‐RBPs were identified with high confidence at TR and RP, respectively, 22 revealed to be dynamic GTS‐RBPs and 30 were seed specific RBPs. Several GTS‐RBPs have been previously reported to play a role in Arabidopsis seed germination. GTS‐RBP EIN2 plays a role in reducing seed dormancy, possibly by repressing the translation of mRNAs that promote dormancy via P‐bodies, while HSP101 and COLD SHOCK PROTEIN 2 (CSP2) promote germination under abiotic stresses (Hong & Vierling, 2001; Koornneef *et al*., 2002; Park *et al*., 2009; Li *et al*., 2015). As mentioned earlier, the GTS is marked by mRNAs that are translationally downregulated (Bai *et al*., 2017). These could be mRNAs that are remnants from maturation, storage proteins or proteins that inhibit germination and thus needs to be degraded (Xu *et al*., 2006). DCP5 may play a role in the decay of these mRNAs via P‐bodies during the GTS especially as the RP as larger granules were observed at this stage (Fig. 4b) (Xu & Chua, 2009). Additionally, several stress granule markers were identified including the TUDOR‐SN protein (TSN1/2) and PAPB2. TSN1/2 has been implied to promote seed germination under salt stress by modulating the mRNA levels of the key GA biosynthesis enzyme *GA20ox3* (Liu *et al*., 2010). Stress granule marker PABP2 formed stress granules after a short heat stress. Other GTS‐RBPs, like APUM5, CSP1 and AtRZ1 have been reported to negatively regulate germination under abiotic stress conditions (Kim *et al*., 2007; Park *et al*., 2009; Huh & Paek, 2014). The presence of RBPs that repress translation or inhibit germination during the GTS, may indicate that during germination, seeds are prepared for quick responses to environmental changes. All together this study provides the first step towards understanding the role of RPBs in the translational control of mRNAs during the GTS, which is important to ensure successful RP and thereby completion of germination.

## Author contributions

NS and LB planned and designed the research. NS, AB, AHPA, LAJW performed experiments. RM provided transgenic lines. NS and LB wrote the manuscript. All authors commented on the manuscript.

## Supporting information


**Fig. S1** Germination curve of the Col‐0 seeds used to determine the GTS time‐points.
**Fig. S2** qRT‐PCR depicting mRNA enrichment after poly‐A pulldown of mRNAs at the radicle protrusion stage of seed germination.
**Fig. S3** Correlation plots between replicates for CL samples at testa rupture and radicle protrusion stages of Arabidopsis seed germination.
**Fig. S4** Confirmation of knockout mutant *at5g47210* using qRT‐PCR.
**Fig. S5** Germination phenotype of *hsp101* under control conditions.
**Fig. S6** Confirmation of dynamic GTS‐RBPs by Western blotting.
**Fig. S7** Visualization of heat stress granule marker PABP2 in Col‐0 and the *hsp101* mutant at testa rupture.

**Table S1** RNA binding proteins identified at (a) testa rupture (TR) and (b) the radicle protrusion (RP) stage of the germination translational shift, including (c) their GO enrichment, (d) protein family classification and (e) overlap with other interactome date sets. In (f) all proteins identified in Input total protein samples at TR and RP are shown.
**Table S2** Dynamic germination translational shift‐RNA binding proteins (GTS‐RBPs) at testa rupture and radicle protrusion during the germination translational shift of seed germination.
**Table S3** Seed specific RNA binding proteins identified by comparison with previously performed interactome captures in *Arabidopsis thaliana*.
Please note: Wiley Blackwell are not responsible for the content or functionality of any Supporting Information supplied by the authors. Any queries (other than missing material) should be directed to the *New Phytologist* Central Office.

## Data Availability

The data that supports the findings of this study are available in the [Link], [Link] of this article.
