# Supplementary material for: The mRNA‐binding proteome of a critical phase transition during Arabidopsis seed germination
Source: New Phytol. 2021 Nov 13;233(1):251–64. doi: 10.1111/nph.17800 (PMC9298696; doi:10.1111/nph.17800)
Supplement: Supplementary file 1 — Fig. S1 Germination curve of the Col‐0 seeds used to determine the GTS time‐points. Fig. S2 qRT‐PCR depicting mRNA enrichment after poly‐A pulldown of mRNAs at the radicle protrusion stage of seed germination. Fig. S3 Correlation plots between replicates for CL samples at testa rupture and radicle protrusion stages of Arabidopsis seed germination. Fig. S4 Confirmation of knockout mutant at5g47210 using qRT‐PCR. Fig. S5 Germination phenotype of hsp101 under control conditions. Fig. S6 Confirmation of dynamic GTS‐RBPs by Western blotting. Fig. S7 Visualization of heat stress granule marker PABP2 in Col‐0 and the hsp101 mutant at testa rupture. [file NPH-233-251-s002.pdf]

New Phytologist

***New Phytologist* Supporting Information**

Article title: Unravelling the mRNA binding proteome at a critical phase transition during Arabidopsis seed germination

Authors: Nikita Sajeev[1], Anirban Baral[1], Antoine H.P. America[2], Leo A.J. Willems[1], Rémy Merret[3] and Leónie Bentsink[1]*

Article acceptance date: 01 October 2021

The following Supporting Information is available for this article:

Fig. S1 Germination curve of the Col-0 seeds used to determine the GTS time-points.

Fig. S2 qRT-PCR depicting mRNA enrichment after poly-A pulldown of mRNAs at the radicle protrusion stage of seed germination.

Fig. S3 Correlation plots between replicates for CL samples at Testa Rupture and Radicle protrusion stages of Arabidopsis seed germination.

Fig. S4 Confirmation of knockout mutant at5g47210 using qRT-PCR.

Fig. S5 Germination phenotype of *hsp101* under control conditions.

Fig. S6 Confirmation of dynamic GTS-RBPs by western blotting.

Fig. S7 Visualization of heat stress granule marker PABP2 in Col-0 and *hsp101* at Testa Rupture.

Table S1a RNA binding proteins identified at testa rupture stage of the germination translational shift

Table S1b RNA binding proteins identified at Radicle protrusion stage of the germination translational shift.

Table S1c GO enrichment analysis based on Molecular function of the GTS and candidate RNA binding proteins identified at Testa rupture and Radicle protrusion stages of Arabidopsis seed germination.

Table S1d Protein family classification of RNA binding proteins at testa rupture and radicle protrusion stages of the germination translational shift.

Table S1e Dataset showing RBPs that are unique and/or overlap between five different interactome captures as shown in Figure 3.

Table S1f Dataset showing all proteins identified in Input total protein samples at TR and RP.

Table S2 Dynamic GTS-RBPs at testa rupture and radicle protrusion during the germination translational shift of seed germination.

Table S3 Seed specific RNA binding proteins identified by comparison with previously performed interactome captures in *Arabidopsis thaliana.*

Fig. S1 Germination curve of Col-0 seeds used to determine the GTS check points. The graph represents the germination percentages at different time-points after the start of imbibition (hours after imbibition, HAI). The testa rupture and radicle protrusion points were chosen when at least 80% of the seeds were at those respective stages. This was determined to be 26 and 42 HAI respectively. The results are representatives of 3 biological replicates (Error bars show standard error).

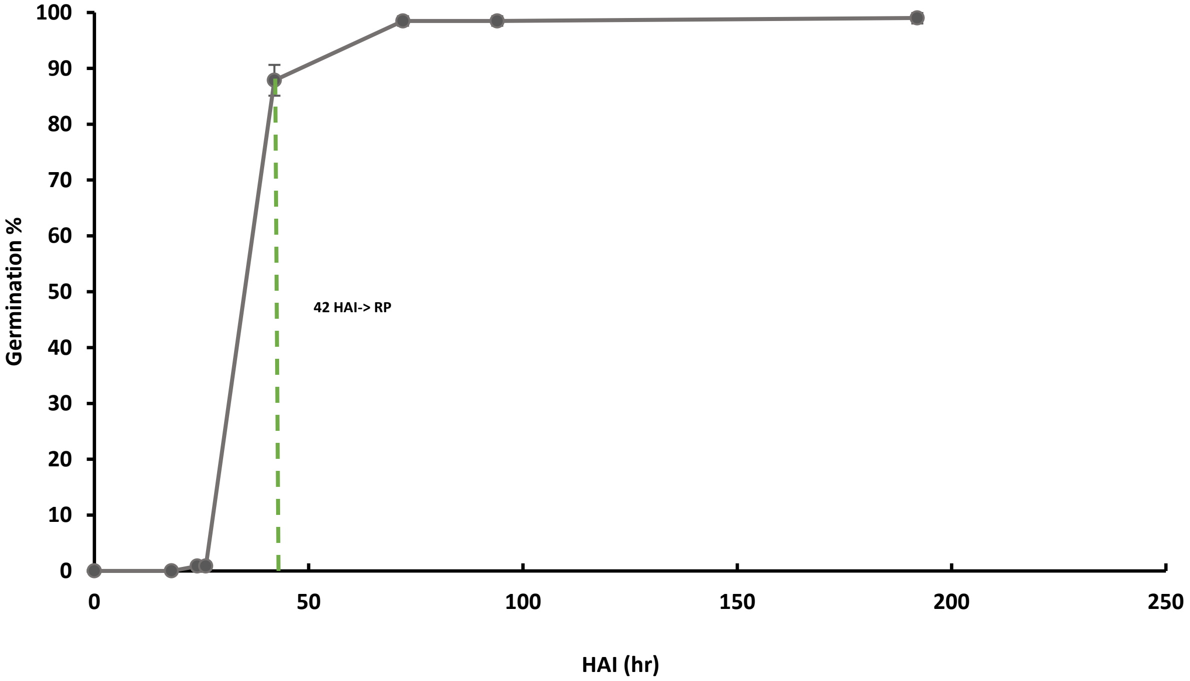

Fig. S2 qRT-PCR depicting mRNA enrichment after poly-A pulldown at the radicale protrusion stage of seed germination. The graph represents the log2 fold change in expression of three mRNAs stably expressed during germination, namely seed housekeeping gene *(HK1)* (*AT4G12590*), *HSP17.6 CII* (AT5G12020) and *PPR related protein* (*AT1G566900*). The relative mRNA expression of the samples after pulldown from both the Crosslinked (CL) and Non-crosslinked controls (NCL) were compared to their total RNA Inputs respectively. Results are representative of 3 biological replicates at the radicle protrusion time point and statistical t- tests were performed. ((*) indicates statistical significance p<=0.05 , error bars show standard error).

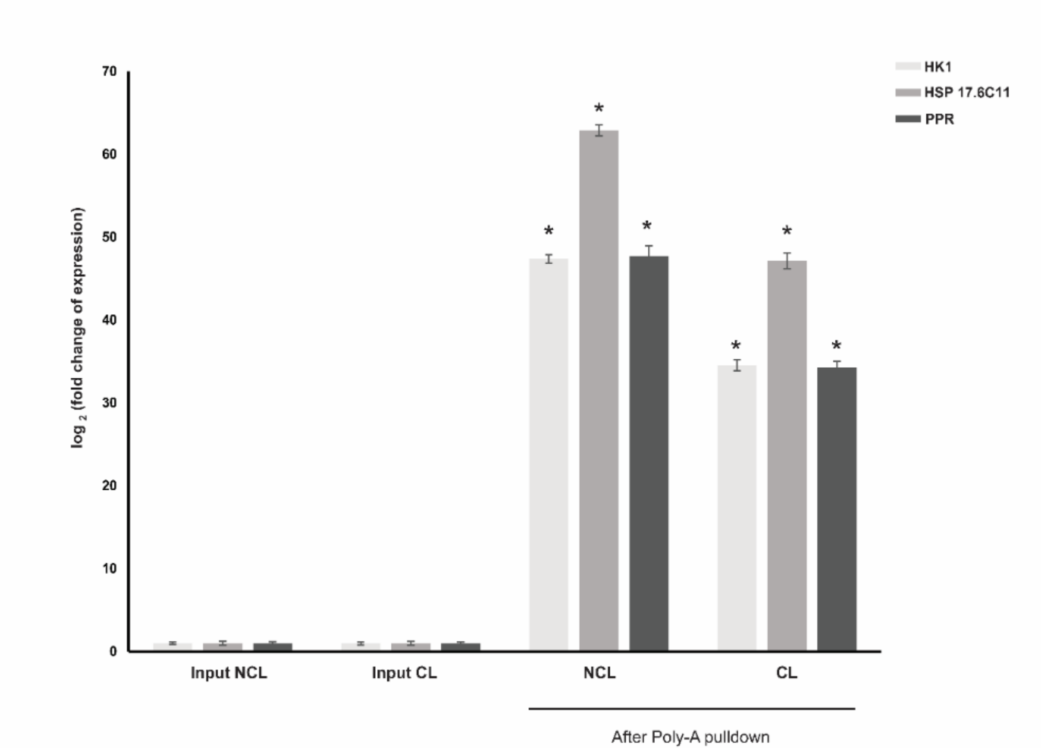

Fig. S3 Correlation plots between replicates for CL samples at TR and RP stages of Arabidopsis seed germination. The correlation plots were made after log2 transformation of the LFQ intensities and imputation of the missing values with constant 10. The graphs correlate the log2 transformed LFQ intensities for the proteins and identified at TR and RP in 3 biological replicates (Rep 1,2 and 3) of the CL samples. R represent the Pearson correlation co-efficient.

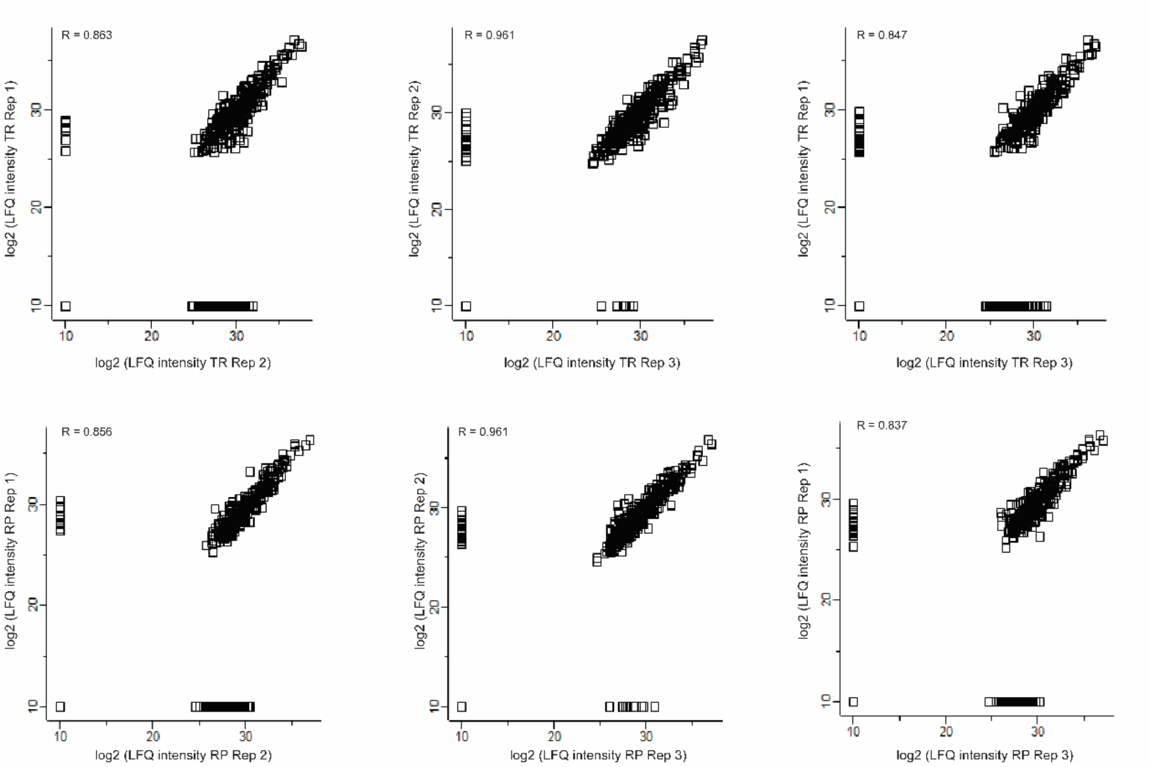

Fig. S4 Confirmation of knockout mutant *at5g47210* using qRT-PCR . The graph represents the log2 fold change in mRNA expression of *AT5G47210* in the mutant *at5g42710* in comparison to the wild type Col-0 in dry seeds and 24 hours after imbibition (HAI). Results are representative of 3 biological replicates at the radicle protrusion time point and statistical t- tests were performed. ((*) indicates statistical significance p<=0.05, error bars show standard error).

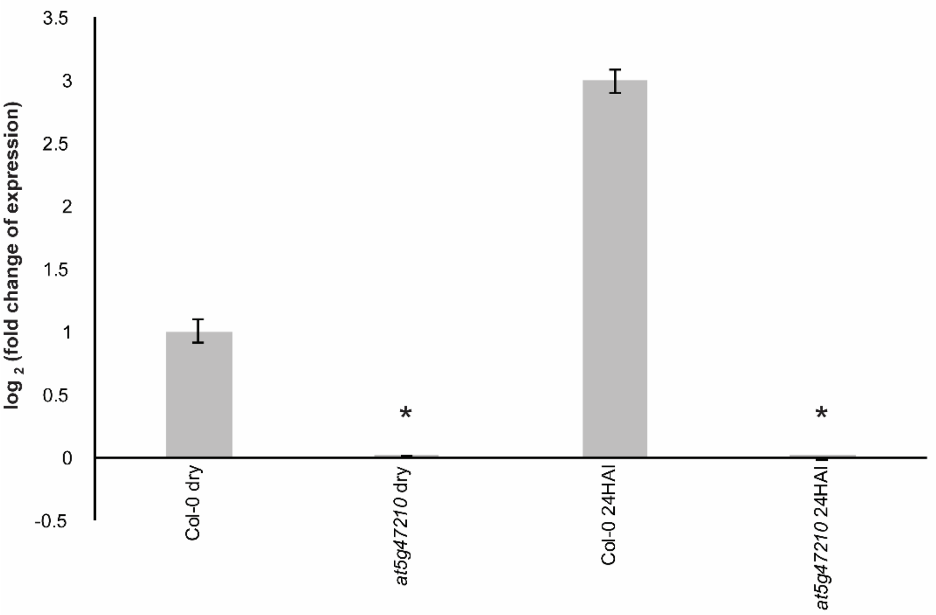

Fig. S5 Germination phenotype of *hsp101* under control conditions: The graphs below represent the maximum germination of (a) dormant freshly harvested seeds (0 days after harvest) and (b) After-ripened seeds (stored for 6 months) of the *hsp101* mutant and complementation line HSP101-RFP in *hsp101* compared to wild-type Col-0 at 22°C. Results are representative of 4 biological replicates and statistical t- tests were performed ($p <= 0.05$, error bars show standard error).

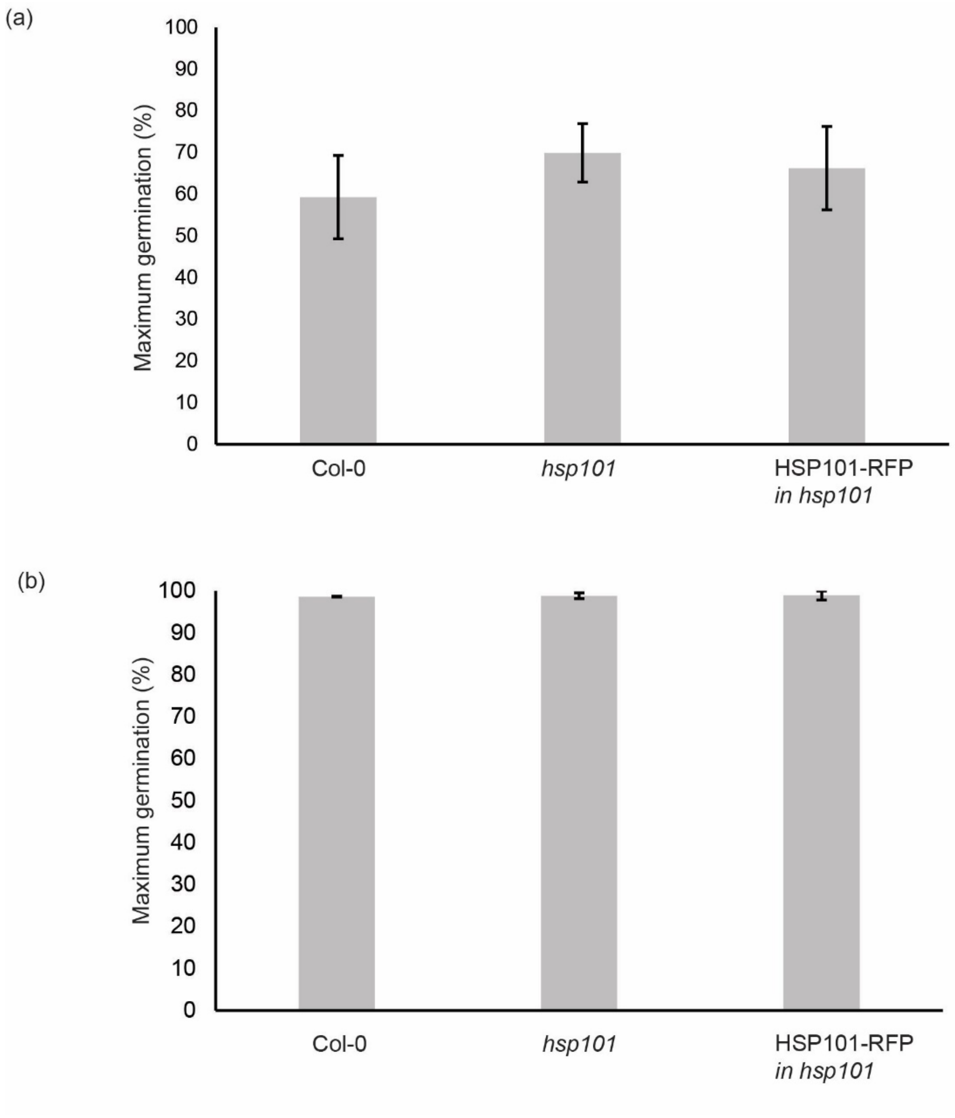

Fig. S6 Confirmation of dynamic GTS-RBPs by western blotting. Western blot images confirming the dynamic nature of GTS-RBPs HSP101 at the Testa rupture (TR) stage and V-ATPase SUBUNIT A at the Radicle protrusion (RP) stage after the poly-A pulldown. AGO1 was used as a known RBP control, while ACTIN 7 as a non-RBP negative control. The non-crosslinked (NCL) and crosslinked (CL) samples were normalized based on the mRNA quantity after the poly-A pulldown, while the total protein input (Inp) samples for TR and RP were loaded with a fixed volume of the total protein from the NCL and CL lysates.

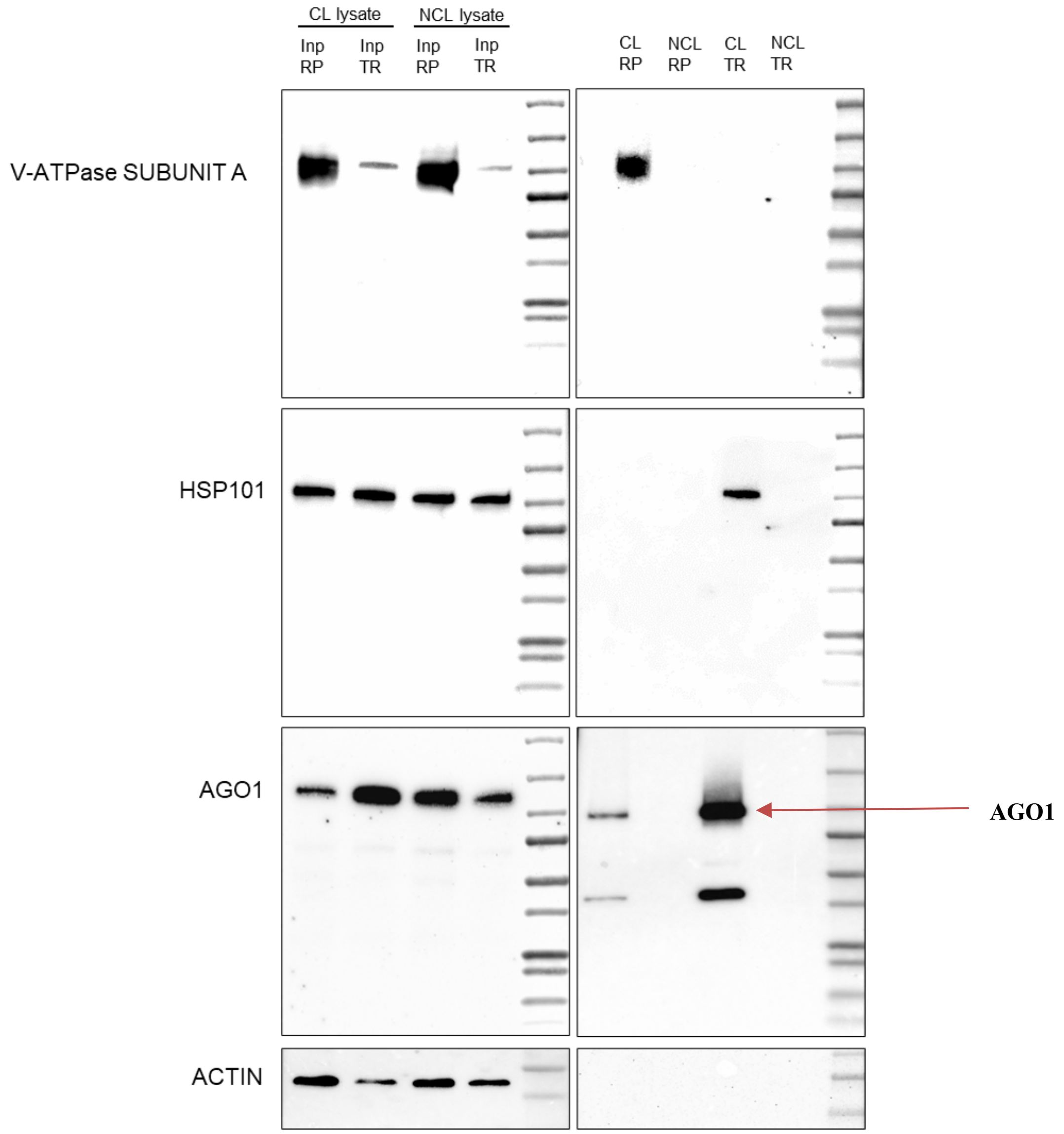

Fig. S7 Visualization of heat stress granule marker PABP2 in Col-0 and *hsp101* at Testa Rupture. Visualizing stress granule formation using reporter line pPABP2-tRFP-PABP2 at testa rupture at optimal germination conditions (control) or under short heat stress of 30 minutes at 42°C. There are no granules observed in control conditions in both wild-type Col-0 and hsp101 seeds at TR. However, after a short heat stress, PABP2 forms significantly more granules in the *hsp101* background compared to the wild type Col-0 as shown in the box-plot. Box-plot showing the number of granules within a diameter range of 20-100 pixels per 1000µm3 volume (n= 30 root epidermal cells and 5 embryos per stage, scale bar = 10 micron, t-test, (∗∗∗) indicates statistical significance p<0.001;). Indicated are the median intensity (middle line), the upper and lower 10th percentile and the whiskers show the range of the data.

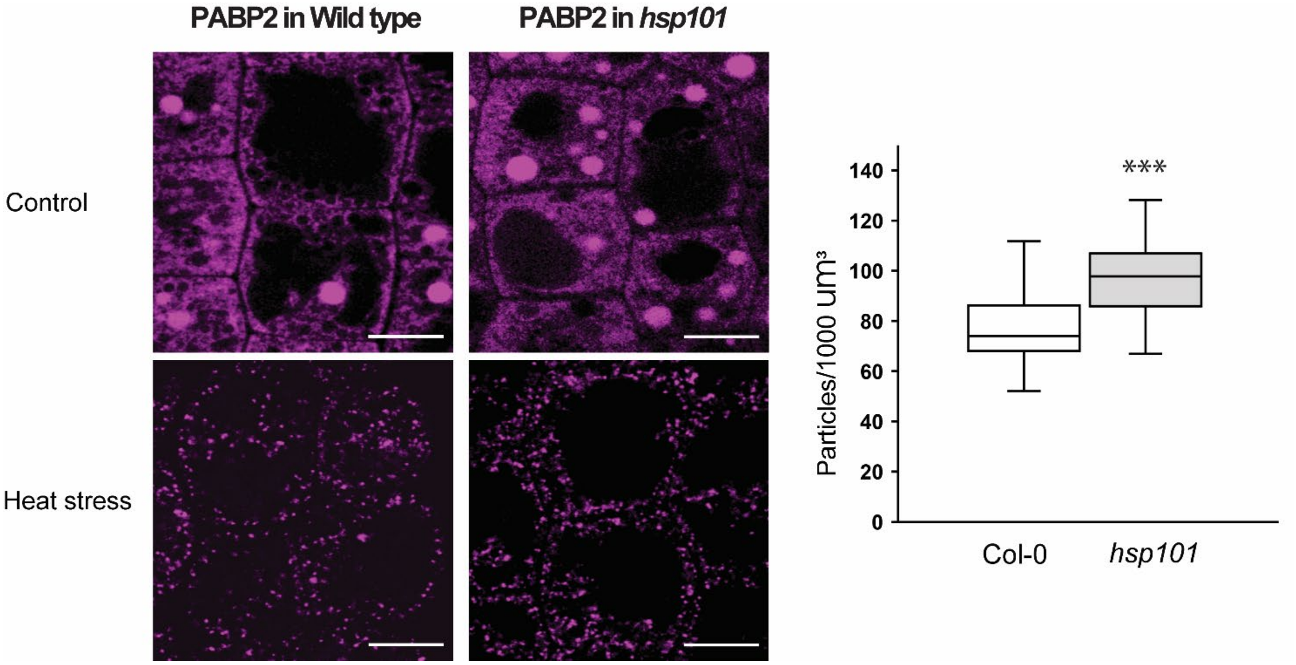

Table S2 Dynamic GTS-RBPs at testa Rupture (TR) and radicle protrusion (RP) during the germination translational shift of seed germination. AGI codes, FASTA headers, the stage at which the protein was more abundant (TR/RP) and whether or not the protein was dynamic in the total input sample are indicated. The proteins that were not identified in the input total protein samples are labelled as 'unknown' in the last column.

**AGI code**	**FASTA header**	**Stage**	**GO Molecular Function**	**Dynamic in Total protein (Input)**
AT4G28440.1	Nucleic acid-binding	TR	mRNA binding	NO
AT2G32080.2	purin-rich alpha 1	TR	mRNA binding	Unknown
AT1G27950.1	glycosylphosphatidylinositol-anchored lipid protein transfer 1	TR	lipid binding	NO
AT1G26110.2	decapping 5	TR	mRNA binding	Unknown
AT5G44320.1	Eukaryotic translation initiation factor 3 subunit 7 (eIF-3)	TR	mRNA cap binding	NO
AT4G10020.1	hydroxysteroid dehydrogenase 5	TR	oxidoreductase activity	YES
AT1G29680.1	histone acetyltransferase (DUF1264)	TR	Unknown	YES
AT1G74310.2	heat shock protein 101	TR	ATP binding	NO
AT3G03710.1	polyribonucleotide nucleotidyltransferase, putative	TR	RNA binding	Unknown
AT3G11710.1	lysyl-tRNA synthetase 1	TR	ATP binding;tRNA binding	Unknown
AT5G03280.1	NRAMP metal ion transporter family protein (EIN2)	TR	mRNA binding	Unknown
AT4G24680.4	modifier of snc1	TR	Unknown	Unknown
AT4G01310.1	Ribosomal L5P family protein	RP	rRNA binding	YES
AT3G14450.1	CTC-interacting domain 9	RP	RNA binding	Unknown

AT4G31580.2	serine/arginine-rich 22	RP	mRNA binding	Unknown
AT3G49390.3	CTC-interacting domain 10	RP	mRNA binding	Unknown
AT5G14040.1	phosphate transporter 3	RP	inorganic phosphate transmembrane transporter activity	NO
AT1G78900.2	vacuolar ATP synthase subunit A	RP	ATP binding	NO
AT3G14415.3	Aldolase-type TIM barrel family protein	RP	oxidoreductase activity	YES
AT2G37340.5	arginine/serine-rich zinc knuckle-containing protein 33	RP	mRNA binding	Unknown
AT4G15530.7	pyruvate orthophosphate dikinase	RP	ATP binding	NO
AT2G18960.3	H(+)-ATPase 1	RP	ATP binding	Unknown

Table S3 Seed specific GTS-RBPs identified by comparison with previously performed interactome captures in *Arabidopsis thaliana*

AGI code	FASTA headers	GO-Molecular Function	Dynamic GTS-RBP	GO-Molecular Function
AT3G14450.1	CTC-interacting domain 9	RNA binding	Yes	RNA binding
AT1G78900.2	vacuolar ATP synthase subunit A	ATP binding	Yes	ATP binding
AT4G15530.7	pyruvate orthophosphate dikinase	ATP binding	Yes	ATP binding
AT2G18960.3	H(+)-ATPase 1	ATP binding	Yes	ATP binding
AT2G01140.1	Aldolase superfamily protein	fructose-bisphosphate aldolase activity	No	fructose-bisphosphate aldolase activity
AT5G62190.1	DEAD box RNA helicase (PRH75)	RNA binding	No	RNA binding
AT1G27950.1	glycosylphosphatidylinositol-anchored lipid protein transfer 1	lipid binding	Yes	lipid binding
AT4G10020.1	hydroxysteroid dehydrogenase 5	oxidoreductase activity	Yes	oxidoreductase activity
AT1G29680.1	histone acetyltransferase (DUF1264)	Unknown	Yes	Unknown
AT1G74310.2	heat shock protein 101	ATP binding	Yes	ATP binding
AT3G11710.1	lysyl-tRNA synthetase 1	ATP binding;tRNA binding	Yes	ATP binding;tRNA binding
AT5G62300.2	Ribosomal protein S10p/S20e family protein	mRNA binding	No	mRNA binding
AT4G09800.1	S18 ribosomal protein	mRNA binding;rRNA binding	No	mRNA binding;rRNA binding

AT2G27530.2	Ribosomal protein L1p/L10e family	RNA binding	No	RNA binding
AT1G56110.1	homolog of nucleolar protein NOP56	snoRNA binding	No	snoRNA binding
AT2G27020.1	20S proteasome alpha subunit G1	endopeptidase activity	No	endopeptidase activity
AT5G27120.1	NOP56-like pre RNA processing ribonucleoprotein	snoRNA binding	No	snoRNA binding
AT1G30580.1	GTP binding	ATP binding;ribosome binding	No	ATP binding;ribosome binding
AT5G07030.1	Eukaryotic aspartyl protease family protein	aspartic-type endopeptidase activity	No	aspartic-type endopeptidase activity
AT1G79690.2	nudix hydrolase homolog 3;nudix hydrolase homolog 3	dipeptidyl-peptidase activity	No	dipeptidyl-peptidase activity
AT3G54400.1	Eukaryotic aspartyl protease family protein	aspartic-type endopeptidase activity	No	aspartic-type endopeptidase activity
AT3G09840.1	cell division cycle 48	ATP binding	No	ATP binding
AT3G15260.2	Protein phosphatase 2C family protein	magnesium-dependent protein serine/threonine phosphatase activity	No	magnesium-dependent protein serine/threonine phosphatase activity
AT1G65090.1	nucleolin	Unknown	No	Unknown
AT5G27640.3	translation initiation factor 3B1	translation initiation factor activity	No	translation initiation factor activity

AT1G24360.1	NAD(P)-binding Rossmann-fold superfamily protein	3-oxoacyl-[acyl-carrier-protein] reductase (NADPH)	No	3-oxoacyl-[acyl-carrier-protein] reductase (NADPH)
AT4G02940.1	oxidoreductase, 2OG-Fe(II) oxygenase family protein	mRNA binding	No	mRNA binding
AT3G57150.1	homologue of NAP57	RNA binding	No	RNA binding
AT5G22470.1	poly [ADP-ribose] polymerase 3	NAD+ ADP-ribosyltransferase activity	No	NAD+ ADP-ribosyltransferase activity
AT2G27040.2	Argonaute family protein	miRNA binding	No	miRNA binding
